# The clinical indexes and immunological status of HIV/AIDS patients undergoing different highly active antiretroviral treatments

**DOI:** 10.3389/fcimb.2024.1436123

**Published:** 2024-12-17

**Authors:** Xinrui Wan, Mingyu Li, Hongye Wang, Ruixian Zhang, Xiaoning Lu, Yu Song, Chenglu He, Renning Zhang, Ming Sun, Hongying Chen, Ya Li

**Affiliations:** ^1^ Department of Clinical Laboratory, The Third People’s Hospital of Kunming, Kunming, China; ^2^ Department of Laboratory Medicine, Yunnan Provincial Institute of Infectious Diseases, Kunming, China; ^3^ Institute of Medical Biology, Chinese Academy of Medical Sciences and Peking Union Medical College, Kunming, China; ^4^ Department of Environmental Health, Yunnan Center for Disease Control and Prevention, Kunming, Yunnan, China; ^5^ Department of Gynecology, The First Affiliated Hospital of Kunming Medical University, Yunnan, China; ^6^ Yunnan Key Laboratory of Laboratory Medicine, Kunming, China; ^7^ Yunnan Province Clinical Research Center for Laboratory Medicine, Kunming, China; ^8^ Department of Clinical Laboratory, The First Affiliated Hospital of Kunming Medical University, Kunming, China; ^9^ Infectious Diseases Department, The First Affiliated Hospital of Kunming Medical University, Yunnan, China

**Keywords:** HIV, HARRT, T-lymphocyte subsets, anemia, liver enzymes

## Abstract

**Objective:**

This study aims to investigate the differences of clinical indices in HIV patients between three different first-line antiretroviral treatment strategies in Yunnan Province, China. Furthermore, the hematologic system, liver function, kidney function, blood lipid levels of HIV patients and its association with CD4+ count, CD8+ count, CD4/CD8 ratio and antiretroviral treatment were also assessed.

**Methods:**

This retrospective cohort study included 81 participants who underwent highly active antiretroviral treatment from September 2009 to September 2019. Baseline sociodemographic and clinical characteristics were collected from each study participant. Routine blood tests, liver and renal function, lipid levels as well as lymphocyte subset counts were measured and recorded for evaluation before and 3, 6, 9, and 12 months after the treatment. Paired t-test was used to compare clinical indices changes after antiretroviral treatment. Univariate linear regression was performed to determine the association between clinical indices and CD4+ count, CD8+ count, CD4/CD8 ratio and antiretroviral treatment.

**Result:**

There were no statistical differences in baseline demographic and clinical characteristics in either treatment group. Compared with the initiation of HARRT treatment, the CD4+ count(p < 0.001), CD4/CD8 ratio(p < 0.001) and PLT(p < 0.001) were increased in the three treatment groups. The TC(p < 0.01) and TG(p < 0.05) were increased in 3TC+AZT+EFV group after treatment. The ALT(p < 0.05), AST(p < 0.01) were decreased in 3TC+EFV+TDF group after treatment. The study indicated statistical differences in CD4+ count (p < 0.001), CD8+ count (p < 0.001), and CD4/CD8 ratio (p < 0.001) in the three treatment cohorts. Furthermore, a strong positive correlation was observed between WBC (p < 0.001), platelet (p < 0.001), Hb (p < 0.001), and CD4+ count in the three treatment cohorts. Moreover, ALT and AST were negatively associated with CD4+ count in the 3TC + AZT + EFV group. Whereas WBC were positively correlated with CD8+ count in the three treatment methods. In addition, platelet and TG were positively correlated with CD8+ count in the 3TC + EFV + TDF. The study also indicated that TC was positively associated with CD8+ count in the 3TC + AZT + NVP group. Furthermore, WBC was negatively related to CD4/CD8 ratio in the 3TC + EFV + TDF group. The platelet level analysis revealed a positive, while TG indicated a negative association with CD4/CD8 ratio in the 3TC + AZT + NVP group. Moreover, ALT and AST were negatively correlated with the CD4/CD8 ratio in the 3TC + AZT + EFV and 3TC + AZT + NVP groups.

**Conclusion:**

The results showed that HIV/AIDS patients treated with different first-line antiretroviral treatment strategies had different hematopoietic, liver, renal and immune system functions. Furthermore, some clinical indicators such as WBC, PLT, TC, TG, and ALT could predict the CD4+ count, CD8+ count, CD4/CD8 ratio levels and recuperation of HIV/AIDS patients, therefore, should be monitored by clinicians.

## 1 Introduction

In the last ten years, highly active antiretroviral therapy (HAART) has been used for the treatment of HIV-infected individuals. However, there are only a few prolonged randomized clinical trials that compare different HAART regimens for immunological outcomes and clinical index (Macarthur et al., 2006). This study has a follow-up time of 10 years, which is longer than other randomized clinical trials comparing three different antiretroviral treatment regimens for clinical index (hematopoietic system liver and renal function) and immunological outcomes. Currently, the most commonly used first-line antiretroviral regimen in China is 2 nucleoside reverse transcriptase inhibitors (NRTIs) + 1 non-nucleoside reverse transcriptase inhibitor (NNRTI) (Sun et al., 2020). The literature suggests that different combinations of antiretroviral drugs have variable effects on immunological responses, hematopoietic system, liver and renal function. However, which combination strategy is required for the treatment of HIV infection is still controversial. CD4+ T and CD8+ T cells are the primary immune response cells in the body, which are closely related to the immune status of HIV-infected patients (Martínez-Sanz et al., 2022). Therefore, CD4+ count after HAART could serve as the main marker for immunological recovery. However, the CD4+ count inadequately reflects immune recovery. The studies have indicated that CD8+ count and CD4/CD8 ratio following HAART are important markers of immune reconstitution (Mussini et al., 2015; Serrano-Villar and Deeks, 2015). In resource-poor areas, due to costs and technical demands, CD4+ count, CD8+ count, and the CD4/CD8 ratio are used to assess immunological recovery and HIV progression (Ojha et al., 2016). However, only a few studies have indicated an association between the CD4+ count, CD8+ count, the CD4/CD8 ratio, the hematopoietic system, and liver function in China. This study aimed to examine the differences in the levels of CD4+ count, CD8+ count, and the CD4/CD8 ratio among various HARRT regimens, and their association with clinical markers levels to the first-line HARRT.

## 2 Methods

### 2.1 Ethics approval

The research protocol of this study was reviewed by the Ethics Committee of the First Affiliated Hospital of Kunming Medical University. Informed consents were acquired from all the participants before they were included in this study. Furthermore, all information and data were validated before analysis.

### 2.2 Sample collection

This retrospective study analyzed 81 HIV/AIDS diagnosed and follow-up patients admitted at the First Affiliated Hospital of Kunming Medical University. The longest follow-up time was 9.9 years, while the shortest was 2.6 years, with a median follow-up time of 5.9 years. The diagnosis of all the patients was confirmed for HIV antibody by the standard method, Western blot, while if required, a nucleic acid test was employed as a supplementary test for HIV load analysis. All confirmed patients were diagnosed following the National TECHNICAL Specifications for HIV/AIDS Testing 2020.Individuals with HIV were eligible for inclusion if they were at least 18 years of age, had at least 24 months’ follow-up on HAART and treated with one of three starting strategies: 3TC + AZT + EFV, 3TC + AZT + NVP and 3TC + EFV + TDF. Individuals who were critically liver, kidney, or other important organ diseases; unable to give responses and blood specimens and had incomplete data records were excluded from the study.

Among the 81 HIV/AIDS patients who underwent HAART from September 2009 to September 2019, 39 patients had CD4+ count ≥ 200 cells/μL and 42 patients had CD4+ count < 200 cells/μL. According to the National Information Management Standards for Free Antiviral Therapy, blood routine tests, liver function, kidney function, and blood lipid levels should be followed up at 3, 6, 9, and 12 months after treatment, as well as free lymphocyte subset counts and viral load analysis once a year should be recorded for analysis and evaluation.

The treatment plan and medical inclusion criteria for patients were implemented following the “National Manual for Free HIV Antiviral Treatment (2^nd^ edition)”. Furthermore, signed “Informed Consent for Free HIV antiviral treatment” was acquired from all the patients, and the drugs were provided free of charge by the state. Adherence was calculated using $\frac{\text{No dose of HARRT taken}}{\text{No of prescribed dose of HARRT}}\times 100\%$ . We considered poor adherence if <85% of doses were taken, and individuals who had poor adherence were excluded from the study.

### 2.3 Treatment method

Of 81 follow-up cases of HIV/AIDS, 28 were treated with lamivudine + zidovudine + Efavirenz (3TC + AZT + EFV) regimen, 35 with lamivudine + zidovudine + nevirapine (3TC + AZT + NVP), 18 with lamivurme + Efavirenz + tenofovir (3TC + EFV + TDF).

### 2.4 Laboratory testing

Fasting venous blood (2 mL) was collected from all the HIV/AIDS patients. FACSCan II flow cytometry (BD Biosciences, San Jose, CA) was performed using the companion reagent CD3/CD4/CD8/CD45 BD Multitest (BD Biosciences, San Jose, CA). Furthermore, the absolute number of lymphocyte subsets was detected and analyzed. All tests were performed within 4 hours after venous blood collection. Moreover, WBC, platelet, and Hb were included in the routine blood test performed *via* the Automatic Hematocyte Counting apparatus (Nisen Meikang, Japan). In addition, TC, TG, ALT, AST, and creatinine levels were assessed to evaluate blood lipid content as well as liver and renal function using Roche Cobas 8000. Samples were prepared using the High Pure System Viral Acid Kit, and the COBAS TaqMan 48 Analyzer was used for automatic amplification and detection. Indoor quality control was performed daily and samples were tested after quality control.

### 2.5 Statistical analysis

All statistical analysis was performed using the IBM SPSS Statistics software package, version 24.0 (IBM Corporation, Armonk, NY, USA). Continuous variables are presented as the mean ± standard deviation (SD). When they conformed to a normal distribution, the differences before and after treatment compared using the paired t-test. Furthermore, the mean of each variable in the 3 cohorts was compared using one-way ANOVA. Categorical variables were presented as frequency and percentage. Comparisons between multiple groups were made using the Chi-squared tests. Moreover, the correlation of CD4+ count, CD8+ count, CD4/CD8 ratio, and clinicopathological variables was assessed via Univariate linear regression. In addition, univariate logistic regression was used to explore the effect of CD4+ count class (CD4+ count ≥ 200 cells/μL or CD4+ count < 200 cells/μL) on these clinicopathological variables. The regression results are presented as regression coefficients (standard error) and *p-values*. A *p-value < 0.05* was deemed statistically significant.

## 3 Results

### 3.1 Baseline demographic and clinical characteristics of patients

Among 81 HIV/AIDS patients who received HAART were enrolled, the baseline characteristics of patients were summarized in **Table 1**. Of the 81 patients, 57 (70.4%) were male and 24 (29.6%) were female; the mean ± SD age was 38.63 ± 2.73 years (range 21-72years). All patients received free standard first-line regimen, 28 (34.6%) were on a regimen of 3TC + AZT + NVP, 35 (43.2%) were on a regimen of 3TC+AZT+NVP, and 18 (22.2%) were on a regimen of 3TC+EFV+TDF. Regarding between groups comparison of baseline demographic and clinical characteristics of patients, there was no statistically significant difference between groups.

**Table 1:** Baseline demographic and clinical characteristics of patients.

Variable		3TC+AZT+EFV	3TC+AZT+NVP	3TC+EFV+TDF	P
All					
		28(34.6)	35(43.2)	18(22.2)	
Sex					
	Male	20(24.7)	24(29.6)	13(16.0)	0.952
	Female	8(9.9)	11(13.6)	5(6.2)	
Age					
	≤38	11(13.6)	15(18.5)	7(8.6)	0.944
	>38	17(21.0)	20(24.7)	11(13.6)	
CD4 cell count at baseline					
	>200	18(22.2)	13(16.0)	8(9.9)	0.095
	≤200	10(12.3)	22(27.2)	10(12.3)	
viral load					
	detectable	3(3.7)	4(4.9)	2(2.5)	0.996
	undetectable	25(30.9)	31(38.3)	16(19.8)	
Exposure group					
	Heterosexual	22(27.2)	30(37.0)	14(17.3)	0.410
	Homosexual	2(2.5)	1(1.2)	1(1.2)	
	IDU	2(2.5)	1(1.2)	3(3.7)	
	Other	2(2.5)	3(3.7)	0(0)	
Marital status					
	Married	19(23.5)	25(30.9)	16(19.8)	0.253
	Other	9(11.1)	10(12.3)	2(2.5)	
Hepatitis B virus status					
	Positive	0(0)	6(7.4)	2(2.5)	0.075
	Negative	28(34.6)	29(35.8)	16(19.8)	
Hepatitis C virus status					
	Positive	3(3.7)	4(4.9)	2(2.5)	0.996
	Negative	25(30.9)	31(38.3)	16(19.8)	

The Categorical variables are presented as frequency and percentage and the frequency for these variables in the 3 groups were compared using Chi-squared tests.

### 3.2 Changes of clinicopathological before and after treatment

**Figure 1:**
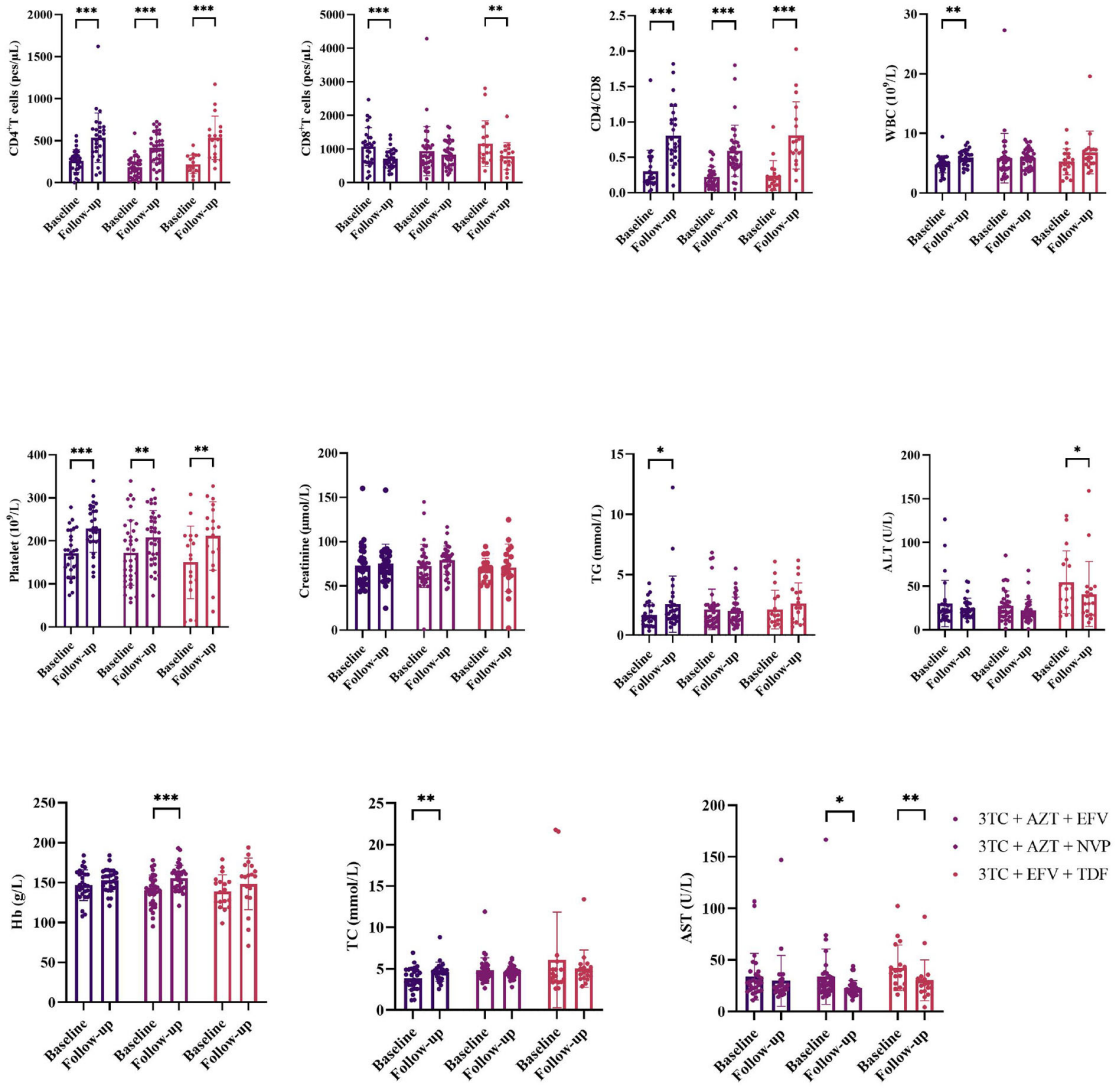
Comparisons changes of clinical indicators before and after treatment. *means p value < 0.05, **means p value < 0.01, ***means p value < 0.005.

After treatment, levels of CD4+ count, CD8+ count, CD4/CD8 ratio, WBC, PLT, TC and TG significantly increased in 3TC + AZT + EFV(CD4+ count:P<0.001; CD8+ count:P<0.001; CD4/CD8ratio:P<0.001; WBC:P=0.004; PLT:P<0.001; TC:P=0.023 and TG:P=0.006). Levels of CD4+ count, CD4/CD8 ratio, PLT, Hb and AST significantly increased in 3TC + AZT + NVP(CD4+ count:P<0.001; CD4/CD8 ratio:P<0.001; PLT:P=0.007; Hb:P<0.001 and AST;P=0.024). Levels of CD4+ count, CD8+ count, CD4/CD8 ratio, PLT, ALT and AST significantly increased in 3TC + EFV + TDF(CD4+ count:P<0.001; CD8+ count:P=0.009; CD4/CD8 ratio:P<0.001, PLT:P=0.003; ALT:P=0.050 and AST:P=0.010), (**Figure 1**).

### 3.3 Descriptive statistics of clinicopathological variables

**Figure 2:**
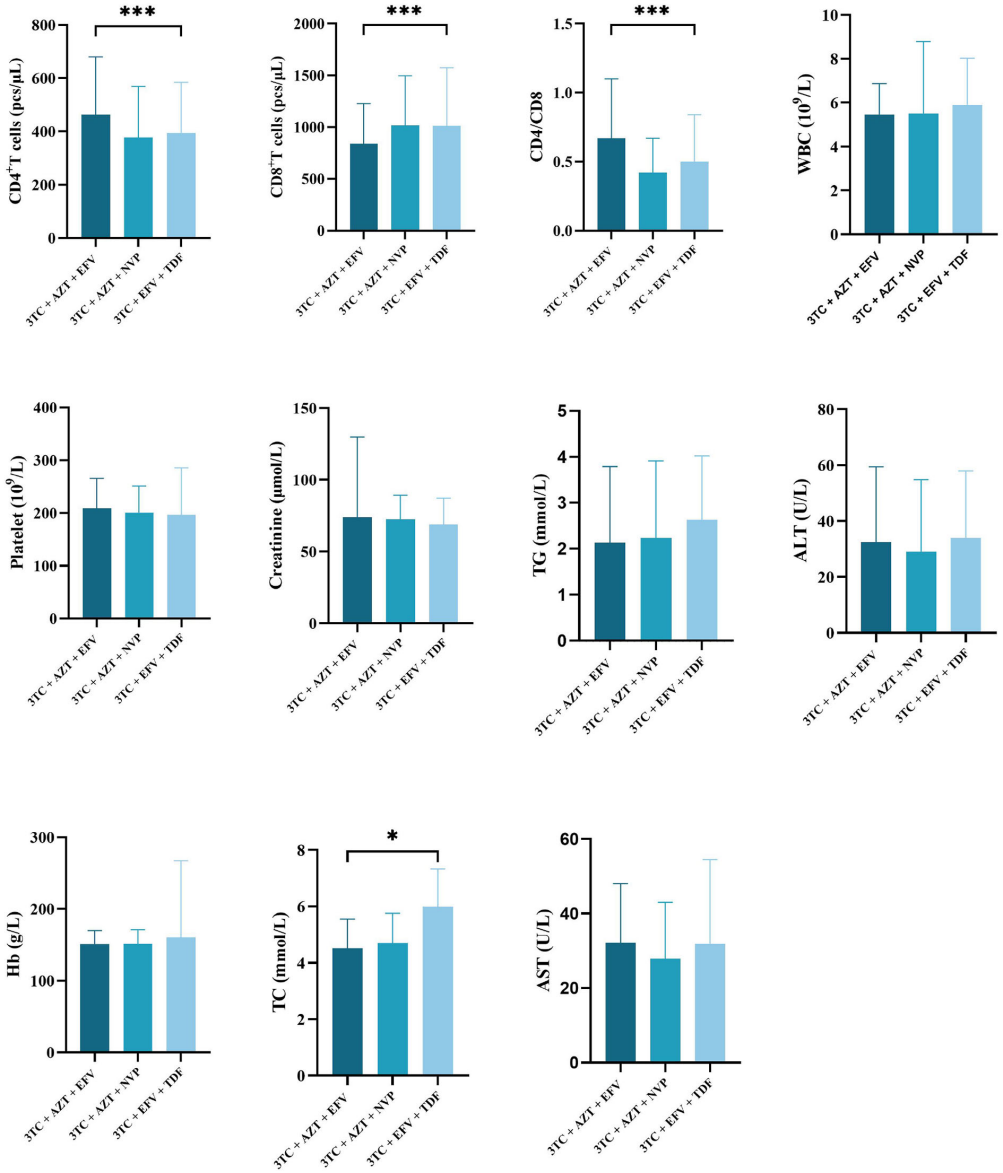
Comparisons differences of clinicopathological variables in three treatment regimen groups. *means p value < 0.05, ***means p value < 0.005.

The differences of CD4+ count (mean ± SD for CD4+ count in 3TC + AZT + EFV, 3TC + AZT + NVP, 3TC + EFV + TDF was 462.24 ± 217.38, 377.30 ± 191.37, and 393.89 ± 190.38, respectively, *p < 0.001*), CD8+ count (mean ± SD for CD8+ count in 3TC + AZT + EFV, 3TC + AZT + NVP, 3TC + EFV + TDF was 839.94 ± 387.12, 1018.24 ± 478.81, and 1014.84 ± 558.94, respectively, *p < 0.001*), and CD4/CD8 ratio (mean ± SD for CD4/CD8 ratio in 3TC + AZT + EFV, 3TC + AZT + NVP, 3TC + EFV + TDF was 0.67 ± 0.43, 0.42 ± 0.25, and 0.50 ± 0.34, respectively, *p < 0.001*) in the three treatment cohorts were significant. Furthermore, significant differences in the TC level were also observed between the three groups (mean ± SD for TC in 3TC + AZT + EFV, 3TC + AZT + NVP, 3TC + EFV + TDF was 4.51 ± 1.04, 4.70 ± 1.06, 5.99 ± 11.34, respectively, *p = 0.019*). However, no differences in WBC, platelet, creatinine, TG, ALT, Hb, or AST were identified between the three groups (**Figure 2**).

### 3.4 Correlation of CD4+ count, CD8+ count, CD4/CD8 ratio and clinicopathological variables

This study indicated a significant positive correlation of treatment days, WBC, platelet, and CD4+ count levels. In the 3TC + AZT + EFV cohort, Beta (SE) for treatment days was 0.106 (0.015) (*p < 0.001)*, for WBC was 49.684 (9.504) (*p < 0.001)*, while for platelet it was 1.003 (0.242) (*p < 0.001*). Furthermore, in the 3TC + AZT + NVP cohort, Beta (SE) for treatment days was 0.073 (0.015) (*p < 0.001)*, for WBC was 11.040 (3.982) (*p = 0.006)*, while for platelet it was 1.236 (0.250) (*p < 0.001).* Whereas in the 3TC + EFV + TDF cohort, Beta (SE) for treatment days was 0.069 (0.020) (*p = 0.001)*, for WBC was 29.100 (6.860) (*p < 0.001)*, while for platelet it was 0.844 (0.159) (*p < 0.001*) (**Table 2**). Moreover, creatinine was negatively associated with CD4+ count in the 3TC + AZT + NVP group [Beta (SE): -2.540 (0.776), *p < 0.001*], ALT was negatively correlated with CD4 in the 3TC + AZT + EFV group [Beta (SE): -1.225 (0.519), *p = 0.019*], while Hb was positively correlated with CD4+ count in the 3TC + AZT + EFV [Beta (SE): 1.848 (0.757), *p = 0.015*] and the 3TC + AZT + NVP [Beta (SE): 1.709 (0.666), *p = 0.011*] groups. Moreover, TC was positively [Beta (SE): 42.087 (13.415), *p = 0.002*], while AST was negatively correlated with CD4+ count in the 3TC + AZT + EFV group [Beta (SE): -2.790 (0.873), *p = 0.002*].

**Table 2:** Univariate linear regression of treatment days, WBC, platelet, creatinine, TG, ALT, Hb, TC, AST, and CD4 grouped by treatment methods.

Variable	3TC + AZT + EFV		3TC + AZT + NVP		3TC + EFV + TDF	
	Beta (SE)	P	Beta (SE)	P	Beta (SE)	P
Treatment days	0.106 (0.015)	< 0.001	0.073 (0.015)	< 0.001	0.069 (0.020)	0.001
WBC	49.684 (9.504)	< 0.001	11.040 (3.982)	0.006	29.100 (6.860)	< 0.001
Platelet	1.003 (0.242)	< 0.001	1.236 (0.250)	< 0.001	0.844 (0.159)	< 0.001
Creatinine	0.325 (0.253)	0.201	-2.540 (0.776)	0.001	-0.799 (0.842)	0.344
TG	-2.381 (8.526)	0.780	-2.226 (2.839)	0.434	1.212 (2.863)	0.673
ALT	-1.225 (0.519)	0.019	-0.799 (0.514)	0.122	-0.196 (0.644)	0.761
Hb	1.848 (0.757)	0.015	1.709 (0.666)	0.011	0.044 (0.144)	0.760
TC	42.087 (13.415)	0.002	-14.346 (12.559)	0.255	-0.594 (1.361)	0.663
AST	-2.790 (0.873)	0.002	-1.764 (0.871)	0.044	-0.605 (0.684)	0.378

In addition, treatment days were negatively correlated with CD8+ count in the 3TC + AZT + EFV [Beta (SE): -0.138 (0.028), *p < 0.001*], 3TC + AZT + NVP [Beta (SE): -0.090 (0.039), *p = 0.022*], and 3TC + EFV + TDF [Beta (SE): -0.240 (0.057), *p < 0.001*] groups (**Table 3**). Furthermore, WBC was positively correlated with CD8+ count in the 3TC + AZT + EFV [Beta (SE): -73.815 (17.222), *p < 0.001*], 3TC + AZT + NVP [Beta (SE): 30.493 (9.922), *p = 0.002*], and 3TC + EFV + TDF [Beta (SE): 119.411 (18.968), *p < 0.001*] groups. Whereas it was observed that platelet was positively correlated with CD8+ count in the 3TC + EFV + TDF group [Beta (SE): 1.014 (0.502), *p = 0.045*]. Moreover, creatinine was positively associated with CD8+ count in the 3TC + EFV + TDF [Beta (SE): 0.971 (0.448), *p = 0.031*] and 3TC + AZT + NVP [Beta (SE): 6.005 (1.948), *p = 0.002*] groups. TG was markedly positively related to CD8+ count in the 3TC + EFV + TDF [Beta (SE): 34.206 (15.022), *p = 0.024*] and the 3TC + AZT + NVP [Beta (SE): 54.043 (6.042), *p < 0.001*] groups, whereas TC was positively associated with CD8+ count in the 3TC + AZT + NVP group [Beta (SE): 119.488 (30.408), *p < 0.001*]. There was no significant correlation of ALT, Hb, or AST with CD8+ count in these groups.

**Table 3:** Univariate linear regression of treatment days, WBC, platelet, creatinine, TG, ALT, Hb, TC, AST, and CD8 grouped by treatment methods.

Variable	3TC + AZT + EFV		3TC + AZT + NVP		3TC + EFV + TDF	
	Beta (SE)	P	Beta (SE)	P	Beta (SE)	P
Treatment days	-0.138 (0.028)	< 0.001	-0.090 (0.039)	0.022	-0.240 (0.057)	< 0.001
WBC	73.815 (17.222)	< 0.001	30.493 (9.922)	0.002	119.411 (18.968)	< 0.001
Platelet	0.353 (0.446)	0.429	0.422 (0.660)	0.523	1.014 (0.502)	0.045
Creatinine	0.971 (0.448)	0.031	6.005 (1.948)	0.002	-2.682 (2.471)	0.279
TG	34.206 (15.022)	0.024	54.043 (6.042)	< 0.001	-5.524 (8.398)	0.512
ALT	0.815 (0.933)	0.383	-0.958 (1.292)	0.460	2.828 (1.877)	0.134
Hb	0.910 (1.363)	0.505	-1.501 (1.690)	0.375	0.386 (0.423)	0.363
TC	44.475 (24.213)	0.067	119.488 (30.408)	< 0.001	-0.325 (3.998)	0.935
AST	0.776 (1.588)	0.625	-0.630 (2.201)	0.775	0.731 (2.011)	0.717

The study also revealed a significant correlation between treatment days and the CD4/CD8 ratio in the 3TC + AZT + EFV, 3TC + AZT + NVP, and 3TC + EFV + TDF groups (all *p-values < 0.001*, **Table 4**). Furthermore, WBC was negatively related to the CD4/CD8 ratio in the 3TC + EFV + TDF group [Beta (SE): -0.027 (0.013), *p = 0.037*]. Whereas platelet was positively linked with the CD4/CD8 ratio in the 3TC + AZT + NVP group [Beta (SE): 0.002 (< 0.001), *p < 0.001*]. Moreover, creatinine and TG indicated a negative correlation with the CD4/CD8 ratio in the 3TC + AZT + NVP group [Beta (SE): -0.005 (0.001), *p < 0.001*] and [Beta (SE): -0.009 (0.004), *p = 0.017*], respectively. ALT and AST were negatively associated with the CD4/CD8 ratio in the 3TC + AZT + EFV [Beta (SE): -0.003 (0.001), *p = 0.004*] and [Beta (SE): -0.005 (0.002), *p = 0.006*], respectively, as well as the 3TC + AZT + NVP group [Beta (SE): -0.002 (0.001), *p = 0.022*] and [Beta (SE): -0.003 (0.001), *p = 0.007*], respectively. No significant correlation of Hb, and TC with CD4/CD8 ratio was observed in these three groups.

**Table 4:** Univariate linear regression of treatment days, WBC, platelet, creatinine, TG, ALT, Hb, TC, AST, and CD4/CD8 ratio grouped by treatment methods.

Variable	3TC + AZT + EFV		3TC + AZT + NVP		3TC + EFV + TDF	
	Beta (SE)	P	Beta (SE)	P	Beta (SE)	P
Treatment days	0.001 (< 0.001)	< 0.001	0.001 (< 0.001)	< 0.001	0.001 (< 0.001)	< 0.001
WBC	0.026 (0.020)	0.191	0.002 (0.005)	0.675	-0.027 (0.013)	0.037
Platelet	0.001 (< 0.001)	0.435	0.002 (< 0.001)	< 0.001	-0.001 (< 0.001)	0.261
Creatinine	-0.001 (0.001)	0.930	-0.005 (0.001)	< 0.001	0.001 (0.002)	0.475
TG	-0.030 (0.017)	0.074	-0.009 (0.004)	0.017	0.005 (0.005)	0.327
ALT	-0.003 (0.001)	0.004	-0.002 (0.001)	0.022	0.001 (0.001)	0.913
Hb	0.002 (0.002)	0.115	0.001 (0.001)	0.846	-0.001 (< 0.001)	0.580
TC	0.024 (0.027)	0.374	-0.020 (0.016)	0.214	-0.002 (0.002)	0.496
AST	-0.005 (0.002)	0.006	-0.003 (0.001)	0.007	-0.001 (0.001)	0.624

### 3.5 Effect of different CD4+ count status on clinicopathological variables

The included participants were categorized into two groups based on the CD4+ count (CD4+ count ≥ 200 and CD4+ count < 200). There was significantly positive linear relationship between CD4 and the CD4/CD8 ratio in the 3TC + AZT + EFV (OR: 33.138, 95% CI: 10.033 - 109.448, *p < 0.001*), 3TC + AZT + NVP (OR: 21.216, 95% CI: 5.601 - 80.361, *p < 0.001*), and 3TC + EFV + TDF (OR: 3.695, 95% CI: 1.333 - 10.238, *p = 0.012*) groups (**Table 5**). Furthermore, There was significantly negative linear relationship between CD4+ count and CD8+ count in the 3TC + AZT + EFV group (OR: 0.999, 95% CI: 0.998 - 1.000, *p = 0.023*). Moreover, There was significantly positive linear relationship between CD4+ count and WBC in the 3TC + AZT + EFV (OR: 1.281, 95% CI: 1.044 - 1.572, *p = 0.017*) and 3TC + EFV + TDF (OR: 1.350, 95% CI: 1.117 - 1.631, *p = 0.002*) groups, whereas CD4+ count was negative linear relationship with creatinine in the 3TC + AZT + NVP group (OR: 0.965, 95% CI: 0.946 - 0.983, *p < 0.001*). In addition, There was significantly negative linear relationship between CD4+ count and ALT in the 3TC + AZT + EFV (OR: 0.977, 95% CI: 0.964 - 0.990, *p < 0.001*) and 3TC + AZT + NVP (OR: 0.981, 95% CI: 0.962 - 0.999, *p = 0.041*) groups, whereas CD4+ count was positive linear relationship with ALT in the 3TC + EFV + TDF group (OR: 1.018, 95% CI: 1.003 - 1.033, *p = 0.015*). CD4+ count was also negative linear relationship with AST in the 3TC + AZT + EFV group (OR: 0.959, 95% CI: 0.939 - 0.979, *p < 0.001*). Other variables (platelet, TG, Hb, and TC) were not affected by CD4 level.

**Table 5:** Univariate logistic regression of treatment days, CD4, CD8, CD4/CD8 ratio, WBC, platelet, creatinine, TG, ALT, Hb, TC, AST, and CD4 class grouped by treatment methods.

Variable	3TC + AZT + EFV		3TC + AZT + NVP		3TC + EFV + TDF	
	OR (95% CI)	P	OR (95% CI)	P	OR (95% CI)	P
Treatment days	1.000 (1.000-1.000)	0.735	1.000 (1.000-1.000)	0.473	1.000 (0.999-1.000)	0.104
CD4	1.004 (1.003-1.006)	< 0.001	1.004 (1.002-1.006)	< 0.001	1.004 (1.002-1.006)	< 0.001
CD8	0.999 (0.998-1.000)	0.023	1.000 (0.999-1.001)	0.986	1.000 (1.000-1.001)	0.260
CD4/CD8 ratio	33.138 (10.033-109.448)	< 0.001	21.216 (5.601-80.361)	< 0.001	3.695 (1.333-10.238)	0.012
WBC	1.281 (1.044-1.572)	0.017	1.178 (0.983-1.410)	0.075	1.350 (1.117-1.631)	0.002
Platelet	1.004 (0.999-1.009)	0.112	1.001 (0.996-1.007)	0.632	1.003 (0.999-1.007)	0.133
Creatinine	0.998 (0.993-1.003)	0.417	0.965 (0.946-0.983)	< 0.001	1.019 (0.998-1.040)	0.077
TG	0.907 (0.775-1.062)	0.226	0.936 (0.803-1.091)	0.397	1.110 (0.962-1.280)	0.152
ALT	0.977 (0.964-0.990)	< 0.001	0.981 (0.962-0.999)	0.041	1.018 (1.003-1.033)	0.015
Hb	0.985 (0.968-1.001)	0.073	1.006 (0.992-1.021)	0.391	0.999 (0.995-1.003)	0.706
TC	0.871 (0.667-1.138)	0.312	0.775 (0.584-1.029)	0.078	0.968 (0.890-1.052)	0.444
AST	0.959 (0.939-0.979)	< 0.001	0.977 (0.953-1.002)	0.072	0.995 (0.976-1.014)	0.574

## 4 Discussion

This study compared three different antiretroviral treatment regimens to assess the long-term clinical and immunological outcomes. The three regimen groups were well balanced in terms of demographic and baseline clinical characteristics. Out of different regimens, no significant difference was observed in terms of gender, age, transmission route, marital status and HBV/HCV status. Besides, the HARRT regimens used in China had shown better response in reducing the HIV-1 viral load, just 9 (11.1%) patients had detectable viral load copies in all three regimen groups. Previous studies have demonstrated that patients with faster reductions in viral load achieve better increases in CD4+ count. The CD4+ count and CD4/CD8 ratio is correlated with the status of immune function of AIDS patients, besides early and maintenance therapy in AIDS patients are important to increase the CD4+ count and CD4+/CD8+ ratio and the immunological reconstitution. This findings show that CD4+ count, CD4/CD8 ratio increased significantly in three HARRT strategies after antiretroviral treatment. The literature suggests that initiating antiretroviral treatment using the following regimens: AZT + 3TC + NVP, AZT + 3TC + EFV, and TDF + 3TC + EFV can prevent treatment failure (Desta et al., 2019). With the growth of age and long-term medication use, multiple complications are increasing, such as hepatic dysfunction, and cardiovascular diseases in AIDS patients. So several clinical indications were compared before and after HARRT treatment. The results showed that PLT was considerably increased in three HARRT regimens, and AST was considerably increased in 3TC + AZT + NVP and 3TC + EFV + TDF groups, besides TC and TG were considerably increased in 3TC + AZT + EFV group. The amounts of platelets after HARRT treatment might be attributed to decreased viral burden and recovered clump of platelets. Long-term use of 3TC and EFV will lead to the elevated transaminases such as ALT and AST. The AZT based regimens come along with higher serum TG levels, that AZT might have a chronic effect on TG regulation *in vivo*.

A significant difference was observed between CD4+ count, CD8+ count, and CD4/CD8 ratios in different HARRT strategies. This study indicated that patients treated with 3TC + AZT + EFV regimen had a significantly increased CD4+ count than those treated with 3TC + AZT + NVP and 3TC + EFV + TDF. Furthermore, CD4+ count are an important index to evaluate the effect of treatment. After antiretroviral treatment, the immune function as well as the volume and function of CD4+ T cells increased. Out of the different regimens, the EFV-based regimen had a higher CD4+ count increment compared to the NVP-based regimen in line with study results from the United States and Uganda (Mwesigire et al., 2015). However, a study from Nepal showed increased CD4+ count after the NVP-based regimen than the EFV-based regimen (Ojha et al., 2016; Sinha et al., 2017). Furthermore, the AZT-based regimen had a significant CD4+ count advantage relative to the TDF-based regimen. However, compared with an AZT-based regimen, a TDF-based regimen has been associated with a higher CD4+ count and a better immunologic outcome (Ayele et al., 2017). Therefore, it was inferred that AZT and EFV based combinations are better than other regimens.

CD8+ count has been associated with inflammatory non-AIDS-related clinical events, and the CD4/CD8 ratio is increasingly recognized as an important marker of immune reconstitution (Lee et al., 2017). This study revealed that the CD8+ count and CD4/CD8 ratio were associated with different antiretroviral treatment regimens. Furthermore, the regimen of 3TC + AZT + EFV lowered the CD8+ count and enhanced CD4/CD8 ratio normalization. Previously, it has been indicated that NNRTI-based regimens were associated with significantly higher rates of CD4/CD8 ratio normalization and decreased CD8+ count (Herrera et al., 2020). Another study indicated increased CD4/CD8 ratio normalization after EFV at the cut-offs of 0.4 and 1.0 (Serrano-Villar et al., 2017). This effect was largely dependent on a greater reduction of CD8+ count observed in the EFV based regimen, the proportion of patients with a CD4/CD8 ratio ≥ 1.0 was higher in the EFV group (Blanco et al., 2018). Moreover, a study investigated the potential influence of the NRTI combination, and patients treated with AZT or 3TC were less likely to normalize their ratio compared with those receiving TDF as backbone NRTIs (Mussini et al., 2015).

To investigate the factors associated with the influence of T lymphocytes among different regimens, the relationship between clinical profile and T lymphocytes was analyzed. It was revealed that the CD4+ and the CD8+ count were increased as treatment days, WBC, and platelet in all three different regimens. HIV also affects the hematopoietic system of the infected individuals which results in cytopenias, that include anemia, thrombocytopenia, and leucopenia (Durandt et al., 2019). Furthermore, the frequency and severity of cytopenias increase with the decline of CD4+ and CD8+ count and enhanced HIV infection (Gebreweld et al., 2020). After the initiation of antiretroviral treatment, the CD4+ and CD8+ count increase, which might be due to the decreased viral load. With increased CD4+ and CD8+ count, a statistically significant increase was observed in WBC and platelet counts (Woldeamanuel and Wondimu, 2018; Vaswani et al., 2022). Moreover, CD4+ and CD8+ T-cell levels were related to WBC, where reduced CD4+ and CD8+ count reduce total WBC counts (Fekene et al., 2018; Bhardwaj et al., 2020). In addition, the Hb of patients with 3TC + AZT + EFV and 3TC + AZT + NVP regimen had increased CD4 T cell count. AZT is the most common among the antiretroviral treatment drugs to cause anemia. The severity of anemia has been correlated with low CD4 count (Suja et al., 2020). Volberding et al. (2003), indicated low CD4+ count in HIV patients with severe anemia.

This study results reveal that the patients on 3TC + AZT + EFV and 3TC + AZT + NVP regimens with elevated ALT and AST had a significantly negative linear relationship with CD4+ count and CD4/CD8 ratio. With the wide use of HARRT, the prevalence of hepatotoxicity among treated HIV/AIDS patients is high. The main reason for HAART-related hepatotoxicity is drug toxicity or drug metabolism, and the observed difference in hepatotoxicity could be associated with the type of HAART regimens (Sterling et al., 2008; Price and Thio, 2010). The liver toxicity of EFV and NVP has been previously described in several studies (Kovari et al., 2016; Wondifraw Baynes et al., 2016). Here, reduced CD4+ count and CD4/CD8 ratio were observed as minor risk factors for liver toxicity. Similar studies have demonstrated that high CD4+ count or CD4/CD8 ratio and NVP or EFV-based regimens are risk factors for hepatotoxicity (Abongwa et al., 2022).

Yu et al. (2023) found that the risk for hypertriglyceridemia was related to both CD4+ count < 200 cells/μL and CD8+ count ≥ 1000 cells/μL in HIV infection. Furthermore, the differences in the CD4/CD8 ratio were significantly greater, and the baseline CD4/CD8 ratio < 0.20 was a risk factor for hypertriglyceridemia. This indicates that the worse the immune state is, the higher the serum TG level will be, which might be because reduced immunity patients are prone to different viral infections, resulting in increased interferon secretion, which in turn elevates the TG levels (Xu et al., 2022). Duan et al. (2022) study has also shown that CD4+ count < 200 cells/μL is a risk factor for hypertriglyceridemia. In different antiviral treatment regimens, there was a significant difference in the relationship between CD8+ count or CD4/CD8 ratio with TG levels in 3TC + AZT + EFV and 3TC + AZT + NVP and 3TC + EFV + TDF. Furthermore, compared with 3TC + TDF + EFV, the other two regimens have differences in AZT. Feeney and Mallon (2011) and Calza et al. (2003) concluded that the use of AZT in antiviral regimens may increase serum TG levels in HIV people. The study investigating the AZT’s effect on serum TG indicated that AZT might have a chronic lesion on TG regulation in the body, just as it produces other toxic side effects (D’andrea et al., 2008). Therefore, the effect of the CD8+ count and CD4/CD8 ratio on TG levels was less significant in the 3TC + TDF + EFV regimen, indicating that for obese or overweight patients, the HARRT regimen has higher efficacy.

Wide HARRT application increases the prevalence of hepatotoxicity among treated HIV/AIDS patients. The main reason for HAART-related hepatotoxicity is drug toxicity or drug metabolism. Furthermore, the observed differences in hepatotoxicity could be associated with the type of HAART regimens. Moreover, the elevated ALT and AST levels had a significantly negative linear relationship with CD4+ count and CD4/CD8 ratio in patients on 3TC + AZT + EFV and 3TC + AZT + NVP regimens. Several studies have assessed the liver toxicity of EFV and NVP previously. In addition, low CD4+ count and CD4/CD8 ratio were identified as a minor risk factor for liver toxicity. Similar studies have demonstrated that low CD4+ count, the CD4/CD8 ratio, and the use of NVP or EFV based regimens are risk factors for hepatotoxicity.

In summary, this study revealed that among different antiviral treatment regimens, patients treated with the 3TC + AZT + EFV regimen had a more favorable immunological effect. It is therefore recommended that the hospital should adopt 3TC + EFV + TDF and 3TC + AZT + NVP as the preferred regimen for patients with anemia and those who need liver protection, respectively.

## Data Availability

The original contributions presented in the study are publicly available. This data can be found here: https://doi.org/10.6084/m9.figshare.27960870.v2.
